# eHealth for service delivery in conflict: a narrative review of the application of eHealth technologies in contemporary conflict settings

**DOI:** 10.1093/heapol/czab042

**Published:** 2021-04-16

**Authors:** Gemma Bowsher, Nassim El Achi, Katrin Augustin, Kristen Meagher, Abdulkarim Ekzayez, Bayard Roberts, Preeti Patel

**Affiliations:** R4HC-MENA, R4HSSS & Conflict & Health Research Group, King’s College London, Strand Ln, London WC2R 2LS, UK; R4HC-MENA, Global Health Institute, American University of Beirut, Beirut 1107 2020, Lebanon; School of Population Health & Environmental Sciences, King’s College London, Guy’s Campus, Great Maze Pond, London SE1 1UL, UK; R4HC-MENA, R4HSSS & Conflict & Health Research Group, King’s College London, Strand Ln, London WC2R 2LS, UK; R4HC-MENA, R4HSSS & Conflict & Health Research Group, King’s College London, Strand Ln, London WC2R 2LS, UK; RECAP, Department of Health Services Research and Policy, London School of Hygiene and Tropical Medicine, London WC1E 7HT, UK; R4HC-MENA & R4HSSS, Dept War Studies, King’s College London, Strand Lane, LondonWC2R 2LS, UK

**Keywords:** Conflict, evidence-based medicine, health planning, health services research, refugee health

## Abstract

The role of eHealth in conflict settings is increasingly important to address geographic, epidemiologic and clinical disparities. This study categorizes various forms of eHealth usage in conflict and aims to identify gaps in evidence to make recommendations for further research and practice. The analysis was carried out via a narrative hermeneutic review methodology. Articles that fulfilled the following screening criteria were reviewed: (1) describing an eHealth intervention in active conflict or ongoing insurgency, (2) an eHealth intervention targeting a conflict-affected population, (3) an e-learning platform for delivery in conflict settings and (4) non-interventional descriptive reviews relating to eHealth in conflict. Of the 489 papers eligible for screening, 46 merited final inclusion. Conflict settings described include Somalia, Sudan, Afghanistan, Syria, Iraq, Pakistan, Chechnya, Gaza and the Democratic Republic of Congo. Thirty-six studies described specific eHealth initiatives, while the remainder were more generic review papers exploring general principles. Analysis resulted in the elucidation of three final categories of current eHealth activity in conflict-affected settings: (1) eHealth for clinical management, (2) e-learning for healthcare in conflict and (3) eHealth for information management in conflict. Obvious disparities in the distribution of technological dividends from eHealth in conflict are demonstrated by this review. Conflict-affected populations are predominantly subject to *ad hoc* and voluntary initiatives delivered by diaspora and civil society organizations. While the deployment of eHealth technologies in conflict settings is increasingly normalized, there is a need for further clarification of global norms relating to practice in this context.

**Key messages**

- The expansion of eHealth technologies has the potential to support the delivery of health services in conflict settings.
- Knowledge and practice gaps exist in this field regarding safety and quality, data privacy and clinical efficacy.
- Further research is required to develop an evidence base to support the further deployment of eHealth resources in these contexts.

## Introduction

The provision and planning for healthcare delivery in conflict is a pressing imperative. Instability, violence and pervasive insecurity have well-documented consequences for the daily running of a health system and for the determination of healthcare burden in affected settings (Martineau *et al.*, 2017). Healthcare within these environments is naturally complex, given the entanglement of affected populations, militaries and oft-deteriorating public services (Ford *et al.*, 2009). As the nature of conflict has evolved in recent decades, so too has the array of digital health tools to address geographic, epidemiologic and clinical disparities in conflict. Digital health technologies, including telemedicine, electronic medical records, wireless health devices (wearables), mobile health (mHealth) and innovative software applications, have the potential to revolutionize healthcare delivery in conflict-affected regions. These tools will become increasingly important in a post-COVID-19 era. The emergence of COVID-19 has seen health systems across the globe expedite digital health methods to provide care in both high- and low-income settings. Methods include telemedicine, epidemiological tracing and public health awareness campaigns through social media, demonstrating their utility as an effective tool to reduce exposure to both patients and healthcare workers. This increasing use of specific eHealth interventions where health systems have been overwhelmed by the prevalence of COVID-19 may provide vital lessons for their applicability in conflict-affected settings.

eHealth is defined broadly as the cost-effective and secure use of information and communications technology (ICT) in support of health and health-related fields, including healthcare services, health surveillance, health literature, and health education, knowledge and research (WHO, 2005; Al Shorbaji, 2008). Such technologies involve a range of system formats; however, eHealth systems typically rely on some combination of synchronous or asynchronous information exchange via text, videoconference, online chat service or telephone conversation (Currell *et al.*, 2001; Cunningham *et al.*, 2014). As the adoption of these technologies has expanded in high-income health sectors, their application has steadily gained prominence in healthcare delivery across conflict-affected regions and stable low- and middle-income settings (Lam and Poropatich, 2008).

It is well established that conflict delivers exceptional challenges for the ordinary provision of urgent and day-to-day healthcare in affected zones (Martineau *et al.*, 2017). Various studies have identified these challenges in contemporary conflicts; a 2012 study by Durrani *et al*. (2012) of the Aga Khan Development Network examining health needs in Afghanistan identifies three categories of health system requirements including (1) needs in provision of care, (2) learning needs and (3) needs in information management. Specific issues identified include human resource shortages, difficulties in referral systems, restrictive government health policies, service utilization obstacles such as healthcare insecurity, lack of continuous health workforce education, lack of access to research, paper-based health information systems and absent inter-professional communication pathways (Durrani *et al.*, 2012; Hill *et al.*, 2014). In recent decades, these challenges have come to intersect with growing technological capabilities specifically in the field of communication and information sharing. Social media, synchronous telecommunication systems, satellite technology and online decision tools have all become commonplace in the health sector (Roine *et al.*, 2001). These eHealth technologies have profoundly changed the practice of healthcare delivery internationally and have been identified by the World Health Organization (WHO) as vital for the development of the field of global public health (WHO, 2017). In addition to high-income settings, significant effort is being directed towards the deployment of these technologies across low- and middle-income settings (LMICs) as a means of reducing programme costs, enhancing health equity and diminishing deficits in outcomes in hard-to-reach and poorly served regions (Perakslis, 2018). Despite the growth of such initiatives in LMICs, there has been limited simultaneous work on the delivery of such services in conflict zones (Woodward *et al.*, 2014; Elamein *et al.*, 2017). System challenges and limited infrastructure provide obvious obstacles; however, as the adoption of information technology outpaces political action, novel forms of intervention via the medium of eHealth technologies have been adopted as part of response programmes delivered by healthcare professionals scattered across the globe. There has been little attention in the academic sphere regarding the application of these technologies in the context of health in conflict, and no reviews to date have been conducted on eHealth interventions in this domain.

This paper seeks to examine the current state of usage of eHealth technologies on healthcare delivery in contemporary conflict settings in order to (1) identify categories of usage and (2) highlight evidence gaps in the application of eHealth in these contexts. Finally, this paper will make recommendations for further research and practice in this field.

## Methods

The analysis was carried out via a narrative hermeneutic review methodology to evaluate the usage of eHealth technologies in conflict (Boell and Cecez-Kecmanovic, 2014). The selection of this methodology over a systematic review was chosen in order to capture a variety of sources, information formats and knowledge bases. The nature of conflict in these settings requires a certain flexibility in public reporting measures due to individual and population safety concerns regarding attribution, technological developments overtaking lengthy scholarly publication cycles and the *ad hoc* nature of many of the reported interventions amidst evolving insecurity. At this relatively early phase of eHealth deployment in disparate conflict zones, a narrative review was felt to best present the heterogeneity of activity whilst drawing out key themes for discursive analysis.

**Figure 1.**
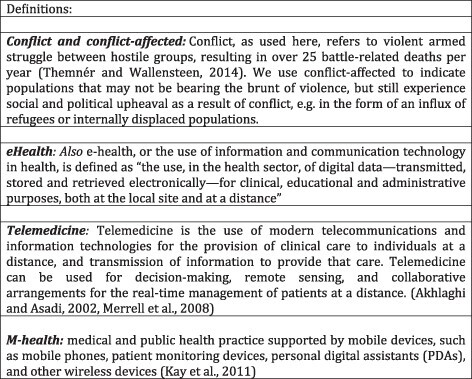
Table of definitions.

Articles were screened according to eligibility as detailed below in the population, intervention, comparison, outcome (PICO) format. Interventions were deemed eligible if they described an eHealth intervention targeting a conflict-affected population—defined for this study as a population experiencing the health consequences of conflict either within a current conflict region during ongoing conflict or insurgency, or as a displaced person from an ongoing conflict. Eligible interventions are eHealth technologies including telemedicine and M-health (defined in Figure 1) deployed in settings of conflict or ongoing insurgency, or with displaced populations from conflict with the goal of improving specific health conditions or healthcare practices (i.e. health information, referral and communication services) either within or outside of the established health sector. Comparison with known health outcomes in specific health domains was made, where such data are readily available. Categorization of the modes of eHealth usage in conflict settings was conducted and recommendations were made for further activity in this domain on the basis of the conclusions.

The literature search examined peer-reviewed articles drawn from the year 2000 onwards using Ovid Medline and Ovid Global Health. Examples of keywords used were telemedicine, eHealth, m-health, digital health, e-learning, conflict, war, insurgency, internally displaced person and refugee. Results were initially screened by title and abstract on the basis of relevance to the subject matter, and remaining studies were read in full before determining the final inclusion. The search was limited to English language literature. A simultaneous grey literature search was carried out using databases including ReliefWeb and the Global Health Observatory (WHO) and the Enterprise Search engine [the United Nations (UN)] as well as the Google search engine to capture media reports and additional open-source documentation. Further suggestions of programmes known to members of the research team were collated. Data were extracted and downloaded onto an Endnote file using a data extraction sheet with key variables including type of eHealth intervention, type of population and region/state of conflict.

Analysis of eligible studies resulted in the identification of three final categories of current eHealth activity in the sphere of conflict. These categories are adapted and expanded from the previously outlined work by Durrani *et al*. (2012) and are defined as (1) eHealth for clinical management, (2) e-learning for health professions training in conflict and (3) eHealth for information management in conflict. Using a grounded coding methodology, the studies were categorized broadly by theme into two phases (1) to establish the format and scope of the described intervention and (2) to localize intervention within one of the three elicited categories described above.

## Results

**Figure 2.**
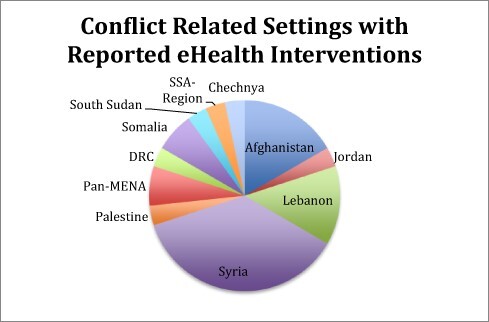
Reported eHealth interventions distributed by conflict.

Of the 489 papers eligible for screening, 46 subsequently merited final inclusion. Conflict settings described included conflicts in various regions encompassing Somalia, Sudan, Afghanistan, Syria, Iraq, Chechnya, Gaza and the Democratic Republic of Congo (DRC). Thirty-six studies described specific eHealth initiatives, whilst the remainder were more generic reviews and discussion papers exploring general principles relevant to eHealth in conflict. The majority of published interventions were reported from within Syria (*n* = 11), with the Middle East and North Africa (MENA) region featuring heavily (*n* = 32). Figure 2 represents the distribution of conflict-related settings with reported eHealth interventions. Studies reported in non-conflict states such as Jordan specifically concern refugee populations.

All studies were published in English. The interventions described incorporated a broad range of interventions from direct clinical management to educational initiatives. Clinical programmes encompassed medical specialties such as neurology, pathology, emergency medicine, plastic surgery, infectious diseases, toxicology, intensive care medicine, dermatology, orthopaedics and mental health interventions.

Assessment of authorship attribution was undertaken to evaluate the location/institutional affiliation of publishing authors. Multiple conflict-affected settings were represented within the authorship including Turkey, Lebanon, DRC, Palestine, Jordan, Afghanistan and Syria. There appeared to be a representation of diaspora scholars affiliated with institutions in the Global North who have strong connections with the affected settings—this was particularly evident for publications relating to the Syrian conflict.

There is a trend towards the provision of Category 1 initiatives, i.e. providing both *ad hoc* and structured eHealth services directly influencing patient care. Tables 1, 2 and 3 represent the categories (1) eHealth for clinical management, (2) e-learning for health professions training in conflict and (3) eHealth for information management in conflict, and the format of reviewed interventions described in the literature.

**Table 1.** Category 1 of eHealth application in conflict

1. Clinical management
SAMS Paediatric TeleITU, Syria (Ghbeis *et al*., 2018)
MSF clinical decision-making tool (Delaigue *et al.*, 2018)
MSF teleneurology service—DRC, Middle East (Saadi and Mateen, 2017)
SAMS ITU, Pulmonary and Sleep Medicine Programme, Syria (Sahloul *et al*., 2016)
Telecardiology, Syria (Alrifai *et al.*, 2018)
Telepsychiatry in Syrian conflict (Jefee-Bahloul, 2014)
IPath telepathology, Afghanistan (Fritz *et al*., 2020)
Teledermatology, Afghanistan (Ismail *et al*., 2018)
Global telemental health—Syrian case study (Jefee-Bahloul *et al.*, 2016)
^a^Telehealth solutions for improving mental health, Afghanistan (Khoja *et al.*, 2016)
^a^eHealth for young adult mental healthcare, Badakshan (Gillis, 2015)
Telemedicine, Middle East hospitals (Patterson *et al*., 2007)
MSF Teleconsultation MDR-TB, the DRC (Shanks *et al*., 2012)
Telemedicine childrens’ hospital, Chechnya (Ehrlich *et al*., 2007)
Teleconsultations, Somalia (Zachariah *et al*., 2012)
Telemedicine, Somalia (Maalim *et al*., 2014)
PASSPORT telepsychiatry, Syria (Jefee-Bahloul *et al.*, 2014)
Teleintensive care, Syria (Moughrabieh and Weinert, 2016)
Telepsychiatry for PTSD, Syria (Nassan *et al.*, 2015)
Teleconsultation cancer care, Syria (Sahloul *et al*., 2017)
MSF Humanitarian Telemedicine Service Inc. CAR, South Sudan (Walji, 2015)
Telemedicine, South Sudan (Joseph, 2013)
eHealth in primary care, Lebanon (Saleh *et al*. 2018a)
M-Health for NCDs in refugee camps (Saleh *et al.*, 2018b)

^a^ Intervention spanning more than one category.

**Table 2.** Category 2 of eHealth application in conflict

2. e-Learning for health professions training
Aga Khan e-learning project, Afghanistan (Durrani *et al.*, 2012)
Healthcare research video conference training, Pakistan (Dodani and LaPorte, 2008)
QMUL Gaza burns education (Theodorakopoulou *et al*., 2019)
RESCAP-MED—NCD policy e-learning platform (Phillimore *et al*., 2019)
International Center for Telemedicine Mobile Emergency Care System (Benner *et al*., 2004)
^a^Telehealth solutions for improving mental health, Afghanistan (Khoja *et al.*, 2016)
Postgraduate burn care education, Gaza (Theodorakopoulou *et al*., 2019)

^a^ Intervention spanning more than one category.

**Table 3.** Category 3 of eHealth application in conflict

3. Health information management
IOM Lebanon Diabetes/HTN information management system (Doocy *et al.*, 2017)
Mental health mobile information app for CHWs, Afghanistan (Khoja *et al.*, 2016, Gillis, 2015)
^a^Telehealth solutions for improving mental health, Afghanistan (Khoja *et al.*, 2016)
^a^eHealth for young adult mental healthcare, Badakshan (Gillis, 2015)
Sijilli: a mobile electronic health records system for refugees in low-resource settings. (Saleh *et al.*, 2019)

^a^ Intervention spanning more than one category.

IOM, International Orgaization for Migration; HTN, hypertension; CHWs, community health workers.

### Category 1: eHealth for clinical management

A minimum of 27 eHealth interventions for clinical management have been identified operating in sites of conflict. Of these interventions, 10 were operative in Syria, 4 in Afghanistan, 2 in the DRC, 3 in a broadly defined ‘Middle East’ with reference to conflict, 2 in Lebanon, 2 in South Sudan, 1 in the Central African Republic (CAR) and 1 in Chechnya. eHealth applications used for clinical management span a breadth of specialities and forms of clinical intervention; these interventions include mental health, dermatology, neurology, intensive care unit (ICU), sleep medicine, cardiology and critical care. Organizations implementing these interventions include the Syrian American Medical Society, Médecins Sans Frontières and academic partnerships with regional ministries of health.

Some interventions lacked detail relating to the conflict context in which these programmes took place. Some Médecins Sans Frontiéres (MSF) programmes, for example, describe deployment in conflict settings without necessarily identifying the precise location of operation. Identification of countries and conflict has been elicited where possible in the results. The true number of conflict sites and programmes deployed may therefore be larger than that described due to vague reporting in the published literature.

### Category 2: eLearning for health professions training in conflict

Seven eHealth interventions directed at health professions education were identified. Some evolved out of necessity rather than initial intention due to changing security contexts such as the European Union–funded RESCAP-MED project, which worked to build research capacity, relevant to public health research, aiming to create a Mediterranean regional network for non-communicable disease (NCD) researchers, in five disciplines: epidemiology, health economics, environmental health, medical anthropology and health policy evaluation. Altered security conditions arising from the Syrian conflict demanded adaptation and the reversion to partial online training offerings rather than planned in-person delivery. Other programmes were designed specifically to overcome specific challenges of the conflict environment for training (e.g. Gaza burns e-learning).

Programmes targeted a varied array of professional groups from medical student teaching, generic healthcare professionals and emergency clinicians (e.g. ICTM emergency care system and health professions training Pakistan) with synchronous and asynchronous teaching products, whilst others addressed specialist clinical domains more prevalent in conflict contexts (e.g. Gaza burns).

### Category 3: eHealth information management in conflict

Five of the reviewed interventions involved the development and delivery of health information management programmes constituting the smallest of the three categories. Three of five interventions were components of larger mental health programmes in Afghanistan and the fourth was a diabetes management programme working with displaced conflict populations in Lebanon.

The nature of these interventions were health records and health data sharing programmes via mobile application formats for use on mobile telephones and tablet devices. All four interventions were sub-components of larger initiatives involving multiple forms of intervention, both in-person and eHealth programmes.

## Discussion

Several interrelated themes emerge from the literature: firstly, the role of eHealth in conflict is an expanding domain for the application of technology in resource-limited contexts. Currently, however, there are obvious disparities in the availability and development of these varied tools. At present, initiatives for eHealth are being produced by predominantly volunteer organizations on an *ad hoc* basis without systematic processes for protocol design and service delivery. Conflict-affected populations do not feature significantly in the literature, suggesting that they are generally excluded from maximizing the medical and technical dividends on offer.

Several diaspora networks and non-governmental organizations (NGOs) are delivering services in constrained environments such as Syria (e.g. Syrian American Medical Society). However, key ethical questions emerge regarding information governance and data protection as clinical data are transmitted via an array of online services with varying degrees of security. A discourse of necessity driving innovation in exceptional circumstances emerges, whereby actors have emerged in the eHealth sphere without a clear mandate for action.

Limited regulatory guidance exists to guide innovations in this field. Impressive programmes delivered by professional diaspora networks delivering remote healthcare services highlight the scope of action in highly contested security environments; however, there is no international consensus on regulation in conflict environments. In a number of described interventions, programmes have emerged as superimposed entities on existing (although often fragmented) services. Current regulatory and accountability mechanisms do not sufficiently address the issue of providers delivering what are often admirably adaptable, but equally *ad hoc* and unregulated services. Contextual issues and the pressures and humanitarian needs of deteriorating conflict conditions drive this phenomenon; however, further policy development to establish minimum standards and regulatory systems are necessary as the technological footprint of eHealth expands in conflict-affected settings. Data privacy too remains a significant ethical challenge for service providers. Personally identifiable information (PII) accessible on digital platforms raises cyber security concerns, which require significant infrastructural and technical investments that may be beyond the scope of some of the small-scale grassroots providers examined in this review. Furthermore, collecting PII in settings of ongoing conflict risks compromising the safety and security of patients and service users whose information might be seized or stolen by parties to conflict and used for non-health objectives.

Customary ethical protocols appear to prevail in the published literature; however, it is unclear where responsibility for oversight lies in many cases. This is despite a plethora of ethical concerns that arise from the use of ICT in healthcare delivery including accuracy and privacy but also the inaccessibility for marginalized members of target communities and cultural or contextual appropriateness (Hunt *et al.*, 2016) (Saleh *et al.*, 2019). The lack of oversight is reflected in a notable absence of UN engagement in conflict-relevant eHealth solutions. Whilst different UN-led programmes and activities focus on using information technology for sustainable development and in disaster response, conflict-affected member states have in the past seen no applicability of eHealth to their settings (UNESCWA, 2011; 2009; 2007). The UN Platform for Space-based Information for Disaster Management and Emergency Response (UN-SPIDER) explicitly rules out involvement of emergencies in armed conflict (UN-SPIDER, 2019) despite their expertise in disaster response and technical and operational issues being equally applicable to conflict situations as to natural disasters (Nicogossian and Doarn, 2011).

Mental health has been at the forefront of the delivery of eHealth technologies in conflict and has frequently been a focus of health partnership activity in these contexts. A partnership between the Aga Khan Foundation, the University of Calgary and a private health technology organization ‘tech4Life’ has designed and disseminated a combined programme of SMS-based mental health inputs for adolescents in Afghanistan, coupled with a community health worker focused mobile application that combines health information capabilities and an e-learning course (Gillis, 2015). In Syria, given severe shortages of psychiatrists (0.5 per 100 000 people) and an estimated burden of 35.5% of population suffering from post-traumatic stress disorder (PTSD), telepsychiatry has been repeatedly proposed as a means of opening up accessibility to treatment (Nassan *et al.*, 2015; Jefee-Bahloul, 2014). In pilot programmes, multiple treatment-resistant cases have been referred for successful treatment via telepsychiatry services (Jefee-Bahloul *et al.*, 2016). However, evaluation of the willingness to participate in such programmes demonstrates a gender disparity with fewer women willing to participate and overall only a 45% willingness to engage in telepsychiatry (Jefee-Bahloul *et al.*, 2014). Reasons motivating a lack of willingness to engage have been described in general terms as a reflection of cultural norms; however, evaluation of specific issues remains to be studied in depth.

The training of health providers working in conflict settings is a field that has received limited specific attention. The Syrian conflict has provided a vehicle for the development of some *ad hoc* initiatives, which show the responsiveness of eHealth technologies to the evolving politics of conflict. The Syrian American Medical Society has, as part of its tele-ICU and cardiology services, delivered structured education services to on-site practitioners in the manner of ward-based teaching and grand round style case-based teaching via WhatsApp and videolink services (Alrifai *et al.*, 2018; Moughrabieh and Weinert, 2016). The Aga Khan-Tech4Life programme for Community Health Workers dealing with mental health concerns in Afghanistan relies heavily on an eHealth component within its mobile application for the ongoing professional development of its staff (Gillis, 2015). This programme emphasizes the scope for integrating eLearning into broader eHealth programmes as part of supporting what the WHO has described as the promise of eHealth for wider health systems development.

Health information management is a central component of the WHO’s health system building blocks (WHO, 2005). In this domain, eHealth has profoundly altered the practice of healthcare in high-income settings; however, sustained benefits are yet to be widely translated across conflict zones. NGOs and civil society organizations, with their attendant IT infrastructure, have led efforts to deliver programmes based on their existing capacities. An intervention delivered by the International Organization for Migration (IOM) and the International Medical Corps worked with Syrian refugee groups in Lebanon to employ a mobile data platform, which served as a patient-controlled electronic health record and a provider decision support tool in the field of hypertension and diabetes prevention. The mobile platform was introduced in primary care settings with the goal of enhancing continuity of care for mobile populations and consequently improving health outcomes. Although satisfaction rates for the initiative were high, there was limited uptake of the tool at the 20-month stage of the programme, suggesting that availability of technology is not the sole obstacle in the delivery of effective eHealth programmes in such settings (Doocy *et al.*, 2017). Given the limited availability of resources for strengthening healthcare systems in conflict, implementing eHealth could be challenging, at a large scale, even if healthcare providers are willing to use it. A 2018 study exploring healthcare provider’s willingness to adopt eHealth interventions in Lebanon identifies major capacity strengthening vacuums requiring attention by policymakers to scale up the use of eHealth in primary healthcare centres ranging from practitioner comfort using computers to absent management support for technological implementation (Saleh *et al.*, 2016).

In the Aga Khan-Tech4Life eHealth interventions in Afghanistan (Khoja *et al.*, 2016; Gillis, 2015), the community health worker mobile application contains a health information recording system with information-sharing capabilities for health data tracking across epidemiologic categories. The use of multi-functional handheld devices possessing health information capacities, rather than large hardware-dependent networks, represents an important reality of conflict settings, where mobile technology is relatively well diffused across resident populations whilst large technical infrastructure is lacking (Collings and Muggah, 2018). Another 2018 Lebanese study demonstrated that using a low-cost e-Health handheld netbook application could facilitate recording of new NCD cases with greater accuracy than existing modelling protocols within rural and refugee settings (Saleh *et al.*, 2018a; 2018b). The gains derived from these disseminated technologies are uncertain in contrast with established and nationally embedded health information systems in other countries; nevertheless, the availability of this information in a structured online format remains a valuable novel resource for various actors working in the constrained operational environments of conflict.

Disruptive technologies in the eHealth field include the expansion of wearable technologies and health self-monitoring systems to monitor health-related metrics as well as aiding techniques such as contact tracing and health promotion through portable devices and mobile applications. Significantly expanding these technologies in fragile and conflict-affected settings is subject to constraint due to obstacles in technological expansion and associated infrastructure, as well as the existing issues of privacy and oversight, which become more germane as the role of portable devices in state surveillance and monitoring systems becomes more visible and contested during the COVID-19 pandemic (Bernard *et al.*, 2020). For the value of such technologies to be realized in a range of contexts, including fragile and conflict-affected zones, further policy-driven work is necessary to adequately address these legitimate concerns.

Community acceptance of online health initiatives is a recurrent challenge raised in the literature. The quality of existing analysis regarding this issue is however limited, and assumptions made regarding the reasons for this are not well evidenced. MSF have suggested some barriers to adoption that broadly encompass cultural, operational and technical issues (Delaigue *et al.*, 2018). The cultural dimension in particular requires more rigorous and sustained examination to appreciate how community and individual perspectives and behaviour interface with changing healthcare delivery systems in a variety of contexts. At this stage, it is challenging to make claims regarding the efficacy of eHealth programmes in conflict settings, despite strong collective sentiment that it is worth developing further. Verifying continuous health outcomes delivered through eHealth programmes and comparing with existing data sets for a range of metrics in conflict-affected settings are important objectives for quality assurance and public health research. An apparent gender disparity in programme uptake requires clearer understanding if eHealth is to be disseminated as part of the earlier described health equity drive. Understanding the availability and uptake of technology is an entry point worth investigating along gender, socioeconomic, age structure and political lines.

Finally, the literature exhibits a notable absence of evidence relating to clinical governance, standards and quality assurance. The assurance of quality in eHealth interventions in conflict settings has not been identified as a key focus in humanitarian response, and scant reporting of safety and quality metrics highlights the lack of sustained focus on maintaining clinical standards according to pre-established criteria. Given the reported uptake of eHealth technologies during the COVID-19 pandemic, this absence requires rapid attention from the humanitarian community working in conflict settings.

## Limitations

The methodology for this review is selected to demonstrate a representative overview of eHealth usage in conflict settings in order to generate thematic information to guide future analysis. Given the current sparse, *ad hoc* and heterogeneous nature of interventions evaluated, it was felt that a systematic review protocol would not generate additional insights beyond the thematic principles already established.

eHealth as a field of practice encapsulates a diversity of technologies and system formats. For the purposes of this study, a broad definition has been adopted and applied to the chosen context of conflict in order to produce an overview of a poorly studied domain. It is understood, however, that as more detailed study takes place, a disaggregation of technology formats will be necessary to address specific questions regarding quality, safety, efficacy, value for money, sustainability, cultural appropriateness and other practical concerns.

## Further research

On the basis of this study, several focused areas for further study emerge. As discussed, there are limited data on the efficacy of such programmes for delivering quality in clinical outcomes in conflict settings. Developing accepted end points for both the technical and clinical components of these initiatives is a necessary step in further expanding their value in these contexts. Comparing technical formats such as SMS, videoconference, web chat and asynchronous platforms in relation to demographic coverage, patient/provider uptake and clinical outcomes is worthwhile for generating an evidence base for the further application of eHealth tools for maximal benefit.

Cost-effectiveness studies are vital on both the patient and provider sides, as well as investigation of the regulatory and information governance factors that promote or limit the expansion of such programmes in complex security environments. In the specific consideration of these factors, further analysis of enabling and disabling conflict dynamics in relation to deployment of eHealth by various types of health actor is required.

## Conclusion

The use of eHealth in conflict presents a clear scope for the delivery of medical care, despite system destruction, health workforce flight and diffuse insecurity. Eliciting the potential of such technologies in a planned manner remains a challenge. Impressive programmes from the conflicts of the 21st century demonstrate the role of technological innovation in addressing escalating health challenges; nevertheless, there remain obvious issues regarding the equitable distribution of the available gains.

The development of a stronger evidence base to justify the widespread deployment of these technologies is required. Conflict dynamics demand specific research as they relate to the application of technology within health programmes. Results from published studies present highly innovative responses to challenging circumstances; nonetheless, published results describe a heavy workflow but equivocal data overall regarding clinical outcomes. Appreciating the complexity of conflict-affected sites for technological distribution in eHealth modalities will require greater understanding of the enabling factors for these technologies. The dissemination of technical capabilities across contested security environments is likely to render persistent operational difficulties—it is therefore essential that information regarding efficacy and cost-effectiveness be made readily available to practitioners and policymakers in this field.
